# Rutin Ameliorates BHBA-Induced Inflammation and Lipid Accumulation in Calf Hepatocytes Through NF-κB Signaling Pathway

**DOI:** 10.3390/cimb47040274

**Published:** 2025-04-14

**Authors:** Kun Yang, Haixia Zhao, Min Gao, Honglian Hu, Dabiao Li

**Affiliations:** 1 College of Animal Science, Inner Mongolia Agricultural University, Hohhot 010018, China; y994454169@163.com (K.Y.);; 2 Institute of Animal Nutrition and Feed, Inner Mongolia Academy of Agricultural & Animal Husbandry Sciences, Hohhot 010031, China; gmyh1588@126.com

**Keywords:** rutin, hepatocytes, BHBA, inflammation

## Abstract

When subclinical ketosis (SCK) occurs in dairy cows, it leads to an excessive production of β-hydroxybutyrat (BHBA), which disrupts liver lipid metabolism and triggers a series of inflammatory responses. Rutin (RT), a flavonoid extracted from plants, exhibits diverse biological activities. However, its potential to mitigate BHBA-induced liver inflammation and lipid accumulation in dairy cows remains unexplored. In this study, we investigated the effect of RT on the BHBA-induced injury of hepatocytes and the possible mechanism. First, hepatocytes were treated with BHBA (0, 0.3, 0.6, 1.2, 2.4 mM) to assess its effects on inflammation impairment and lipid accumulation. Second, hepatocytes were pretreated with RT (0, 25, 50, 100, 150 μg/mL) to evaluate its protective effects. Third, hepatocytes were divided into five treatment groups: blank control, BHBA treatment, RT + BHBA treatment, NF-κB activator (PDTC) + BHBA treatment, and RT + PDTC + BHBA treatment. This experiment further explored the underlying mechanism of RT in mitigating BHBA-induced hepatocyte injury. The results demonstrated that RT at 100 and 150 μg/mL mitigated the increases in hepatocyte interleukin-1 beta (IL-1β), IL-6, triglyceride (TG), and total cholesterol (TC) contents induced by high concentrations of BHBA (*p* < 0.05). Compared to the BHBA treatment, 100 μg/mL RT significantly downregulated the relative protein expression of P-NF-κB p65 and the relative mRNA expression of NF-κB p65, tumor necrosis factor-alpha (TNF-α), IL-1β, IL-6, peroxisome proliferator-activated receptor gamma (PPARγ), and microsomal triglyceride transfer protein (MTP), while upregulating the relative mRNA expression of IKBα (*p* < 0.05). Additionally, these effects were more pronounced with the combined pretreatment of the PDTC and RT. In conclusion, RT inhibits BHBA-triggered hepatocyte inflammation and lipid accumulation by modulating the NF-κB signaling pathway, implying that RT may be a promising target for ameliorating damage in SCK cows.

## Simple Summary

Rutin protects and stabilizes hepatocytes and is used to treat hepatitis, liver cirrhosis, and other diseases. However, there is little evidence to suggest that rutin is associated with BHBA-induced hepatotoxicity. In this study, we investigated the effects of rutin on BHBA-induced liver inflammation and lipid accumulation in calf hepatocytes and discussed the in vitro mechanisms.

## 1. Introduction

SCK is a common metabolic disorder in high-producing dairy cows, particularly during the periparturient period. As calving approaches, cows must meet increasing energy demands to support both the rapidly growing fetus and lactation. However, during the prepartum period, dry matter intake (DMI) gradually declines despite the heightened need for energy and nutrients, resulting in a state of NEB [1,2]. To compensate for this energy deficit, cows enhance lipid metabolism, leading to the rapid mobilization of fat and the release of large amounts of free fatty acids (FFA). A portion of these FFAs is transported to the liver via the bloodstream, where they are either oxidized for energy production or converted into TG. Excessive TG accumulation can lead to fatty liver disease in severe cases [3,4]. Additionally, some FFAs undergo incomplete oxidation, generating ketone bodies, predominantly BHBA [5]. BHBA is acidic in the blood, and prolonged elevation beyond physiological thresholds can trigger inflammatory responses and metabolic acidosis. Moreover, excessive BHBA accumulation in circulation leads to SCK, which is difficult to detect in its early stages. If left unmanaged, SCK can progress to clinical ketosis, causing significant economic losses to dairy farms [1]. Wang et al. demonstrated that during NEB, cows produce elevated levels of BHBA, which significantly downregulate the mRNA expression of the fat catabolism gene PPARα, while Oil Red O staining reveals extensive lipid accumulation in liver tissue [4].

RT is a purely natural flavonoid compound widely found in the roots, stems, leaves, and fruits of plants, and it has antitumor, antibacterial, antioxidant, anti-inflammatory and other biological activities [6,7]. Flavonoids such as quercetin have been shown to exert protective effects in dairy cattle. For instance, quercetin alleviates lipopolysaccharide (LPS)-induced inflammatory damage and ferroptosis in bovine hepatocytes [8]. Duan et al. [9] demonstrated that quercetin mitigates hydrogen peroxide-induced oxidative stress in bovine follicular cells. Similarly, Dai et al. [10] reported that dietary supplementation with Scutellariae radix flavonoid extract enhances antioxidant capacity, immune function, and productive performance in dairy cows. NF-κB is a transcription factor that plays a critical role in regulating apoptosis and inflammation [11]. Wang et al. demonstrated that RT alleviates zearalenone-induced hepatic inflammatory injury in mice by inhibiting the NF-κB signaling pathway [12]. Similarly, He et al. showed that RT significantly decreased IL-1β and IL-6 contents, increased IL-10 contents, and inhibited NF-κB expression in hepatocytes, thereby mitigating carbon tetrachloride-induced inflammatory injury [13]. Moreover, RT was found to regulate short-chain fatty acid metabolic pathways, inhibit fat accumulation, improve disorders of glucose and lipid metabolism, and reduce lipid-induced damage [14]. However, there is little evidence to suggest that RT is associated with BHBA-induced hepatotoxicity. Based on these findings, we hypothesized that RT protects calf hepatocytes from BHBA-induced oxidative stress and inflammatory damage by regulating the NF-κB pathway. Therefore, this study aimed to investigate whether high concentrations of BHBA induce inflammatory injury and lipid accumulation in dairy cow hepatocytes and to determine whether RT exerts a protective effect against BHBA-induced hepatocyte inflammation and lipid accumulation, while elucidating its underlying mechanisms. The findings of this study may provide a theoretical basis for the prevention and treatment of SCK in dairy cows.

## 2. Materials and Methods

### 2.1. Calf Primary Hepatocyte Culture

All Holstein calves used in this study were strictly cared for according to the principles of the Institutional Animal Care and Use Committee (IACUC) of Inner Mongolia Agricultural University (Approval No. NND2022049, on 22 March 2022). Three newborn healthy female Holstein calves (1 d old, female, 30–40 kg, fasting) were selected as hepatocyte donor animals from a large-scale farm in Changchun City, Jilin Province, China. Primary hepatocytes were isolated using a two-step collagenase perfusion method, as previously described [15,16]. This technique enables the stable and efficient acquisition of a large number of calf primary hepatocytes. Briefly, the scalpel was used to obtain the caudate lobe of liver from the calf. The interior of the caudate was flushed with prewarmed solution A (140 mM NaCl, 0.5 mM EDTA, 6.7 mM KCl, 10 mM HEPES, and 2.5 mM glucose; pH 7.2–7.4) at 37 °C until it was clarified (perfusion time of approximately 15 min at a flow rate of 50 mL/min), and then the interior of the caudate of the liver was flushed with solution B (140 mM NaCl, 30 mM HEPES, 6.7 mM KCl, 5 mM CaCl_2_, and 2.5 mM glucose, pH 7.2–7.4) at 37 °C until thoroughly clarified. Subsequently, livers were digested with perfusion solution C (0.1 g collagenase IV dissolved in 0.5 L of perfusion solution B, pH 7.2–7.4, 37 °C), and the flow rate was reduced appropriately to allow for adequate digestion (20 mL/min for 20 min). Digestion continued until the liver tissue was soft and fluffy as a flocculent and the digested effluent was slightly cloudy. Fetal bovine serum (FBS; Hyclone Laboratories) was then added to terminate the digestion. Blood vessels, fat, and undigested liver tissue were removed, subsequently washed twice in RPMI-1640 basic medium (RPMI-1640 medium supplemented with 2% bovine serum protein; BSA; B2064, Sigma-Aldrich Co, St. Louis, MO, USA), centrifuged for 5 min at 500× *g* at 4 °C, and then filtered 2 times with 100 mesh and 200 mesh cellular filters. The cell density was adjusted to 2 × 10^6^ cells/mL using RPMI 1640 adherence medium (to 250 mL of RPMI 1640 medium, 10% FBS, 1 mM bovine insulin 230 μL, 1 mM dexamethasone 8 μL, 5 mg/mL vitamin C 4 μL, and penicillin–streptomycin at a final concentration of 1% were added). After 4 h, the hepatic cells began to adhere and spread, and the medium was replaced with RPMI-1640 complete medium (RPMI-1640 medium supplemented with 10% FBS). Afterwards, the solution was changed every 24 h, observing the morphology of the hepatic cells using an inverted microscope (ECLIPSE Ti2). Cell viability, assessed using trypan blue exclusion, was greater than 95%, meeting the criteria for subsequent experimental procedures.

### 2.2. BHBA and RT Preparation

We weighed 1.206 g of BHBA (P0500, Sigma Aldrich) and dissolved it in 50 mL of H_2_O, filtered through a 0.22 μm sterile filter for sterilization to a final concentration of 0.2 mM, stored in a −20 °C refrigerator for later use. RT (B20500; >95%, HPLC) was purchased from Shanghai Yuanye Biotechnology Co., Ltd. (Shanghai, China). RT (20 mg) was dissolved in 0.328 mL of dimethyl sulfoxide (DMSO, Sigma Aldrich) to generate a 100 mM concentrated stock solution of RT.

### 2.3. Experimental Design

Firstly, the CCK-8 assay was used to assess cell viability under different BHBA exposure times. To investigate whether BHBA induces damage and lipid accumulation in hepatocytes, we treated the cells with 0, 0.3, 0.6, 1.2, and 2.4 mM BHBA. Secondly, to investigate the effect of BHBA on hepatocyte inflammatory factors and the intervention of RT, as well as the impact of RT on BHBA-induced lipid accumulation in hepatocytes, cells were pre-treated with 0, 25, 50, 100, or 150 μg/mL RT for 24 h, and then treated with 1.2 mM BHBA for 24 h. In addition, hepatocytes were stained with Oil Red O to observe lipid accumulation. Thirdly, to investigate whether RT can alleviate BHBA-induced hepatocyte inflammation and lipid metabolism injury via the NF-κB signaling pathway, we established five treatments: blank controls; BHBA treatment; RT + BHBA treatment; PDTC + BHBA treatment; RT + PDTC + BHBA treatment. In the BHBA treatment, cells were treated with 1.2 mM BHBA for 24 h. In the RT + BHBA treatment, cells were protected with 100 μg/mL RT for 24 h and then treated with 1.2 mM BHBA for 24 h. In the PDTC + BHBA treatment, cells were pre-treated with 10 μM PDTC for 2 h and then treated with 1.2 mM BHBA for 24 h. In the RT + PDTC + BHBA treatment, cells were pre-treated with 100 μg/mL RT for 24 h and 10 μM PDTC for 2 h, and then treated with 1.2 mM BHBA for 24 h. Each experiment was repeated at least six times.

### 2.4. Cell Viability

Cell viability was assessed with a CCK-8 kit (Solarbio Life Sciences, Beijing, China), according to the manufacturer’s instructions. A total of 5000 cells were inoculated into each well of a 96-well plate and incubated at 37 °C in 5% CO_2_. Hepatocyte fusion was observed to be approximately 90% under an inverted microscope. A final concentration of 1.2 mM BHBA was added and incubated for 0, 6, 12, 24 and 48 h, respectively. Both blank and control groups were established and washed with 1× PBS twice, and 10 μL of CCK-8 solution was added to each well and then incubated for 3 h at 37 °C in 5% CO_2_. Subsequently, the absorbance values of each treatment group were measured at 450 nm using a microplate reader (Thermo Fisher Scientific, Waltham, MA, USA).

### 2.5. Detection of Inflammation Indicators

The contents of TNF-a, IL-1β, IL-6, and IL-10 were measured using bovine ELISA assay kits (Angle Gene Co., Ltd., Nanjing, China), according to the manufacturer’s instructions.

### 2.6. TG, TC Determination, and Oil Red O Staining

The contents of TG and TC were measured using biochemical kits (Angle Gene Co., Ltd.), according to the manufacturer’s instructions, with detection limits ranging from 0.1 mmol/L to 5 mmol/L. Oil Red O staining was performed according to the protocol provided in the Oil Red O Staining Kit (Solarbio, Beijing, China). In brief, hepatocytes were rinsed twice with PBS and fixed with 4% formaldehyde for 10 min. After fixation, the cells were rinsed twice with distilled water and stained with Oil Red O for 10 min. The stained cells were then washed with 60% isopropanol and rinsed twice with distilled water. Next, the cells were counterstained with Mayer’s hematoxylin, followed by a 3 min rinse with distilled water. Finally, the stained cells were imaged using a fluorescence microscope (IX73, Olympus, Tokyo, Japan).

### 2.7. Real-Time Quantitative PCR Assay

Briefly, total RNA was extracted from cells, according to the TRIzol (Invitrogen Corporation, Carlsbad, CA, USA) manufacturer’s instructions. Total RNA (1 µg) was reverse-transcribed into cDNA using M-MLV Reverse Transcriptase (RNase H-; TaKaRa Biotechnology Co., Ltd., Otsu, Japan), according to the manufacturer’s protocol. The reaction was carried out using a 20 μL reaction system. qRT-PCR was conducted using the FastStart Universal SYBR Green Master (ROX; 04913914001, Roche, Basel, Switzerland) with the BioRad iCycler iQTM Real-Time PCR Detection System (BioRad Laboratories Inc, Hercules, CA, USA). The reaction system had a volume of 20 μL, with five replicates per sample. The reaction conditions were as follows: initial denaturation at 95 °C for 1 min, followed by 40 cycles of 95 °C for 15 s and 63 °C for 25 s, with fluorescence collected at each cycle. The expression levels of NF-κB p65, inhibitor of nuclear factor kappa B alpha (IKBα), TNF-α, IL-1β, IL-6, PPARγ, and MTP were measured, with β-actin (ACTB) used as the endogenous control gene. Target gene mRNA abundance was calculated using the 2^−ΔΔCT^ method. Gene primers are shown in Table 1. Each treatment was performed in six replicates.

### 2.8. Protein Extraction and Western Blotting Assay

The cell precipitates were collected, and the total proteins from calf primary hepatocytes were extracted according to the protocol provided with the Total Protein Extraction Kit (containing Protease Inhibitor Cocktail). The extracted proteins were then quantified using the BCA Quantification Kit. Equal amounts of protein (10 μg per lane) were loaded onto 10% SDS-PAGE gels for separation and subsequently transferred to polyvinylidene difluoride (PVDF) membranes (IPVH00010, Millipore, Burlington, MA, USA). After membrane transfer, the transferred membranes were washed twice with Tris-HCl buffer solution (T-TBS). They were then blocked in TBS containing 5% skimmed milk powder for 1 h at room temperature. Primary antibodies were dissolved in T-TBS containing 3% skimmed milk powder and diluted separately at 1:1000: p-NF-κB p65 (Bioss, Beijing, China), NF-κB p65 (Cell Signaling Technology, Danvers, MA, USA), β-actin (Santa Cruz Biotechnology, Dallas, TX, USA). The PVDF membranes were incubated with the primary antibodies at 4 °C overnight. After incubation, the membranes were washed three times with T-TBS for 5 min each. Subsequently, the membranes were incubated with the secondary antibody (goat anti-rabbit IgG, Beyotime Biotechnology, Haimen, China) diluted 1:4000 in blocking buffer for 1 h at room temperature. Finally, the membranes were washed five times with T-TBS, with each wash lasting 5 min. The blots were visualized using an enhanced chemiluminescence (ECL) detection system (Beyotime Biotechnology). The relative intensities of the protein bands were quantified using ImageJ Quantity One 1-D Version 4.6.8 (National Institutes of Health) and normalized to β-actin levels. Each treatment was performed in six replicates.

### 2.9. Statistical Analysis

All data were analyzed using SPSS 22.0 (SPSS Inc., Chicago, IL, USA) software. The normality of all parameters was assessed using the Shapiro–Wilk test. Data were presented as means ± standard error of the mean (SEM). For normally distributed data from calf hepatocytes, statistical comparisons were made using a two-tailed unpaired Student’s *t*-test or one-way ANOVA followed by Bonferroni correction. For non-normally distributed data, the Mann–Whitney U test was applied. *p* < 0.05 was considered statistically significant, while *p* < 0.01 was considered highly significant.

## 3. Results

### 3.1. Effect of BHBA on Cell Viability and Inflammatory Indices in Calf Hepatocytes

**Figure 1:**
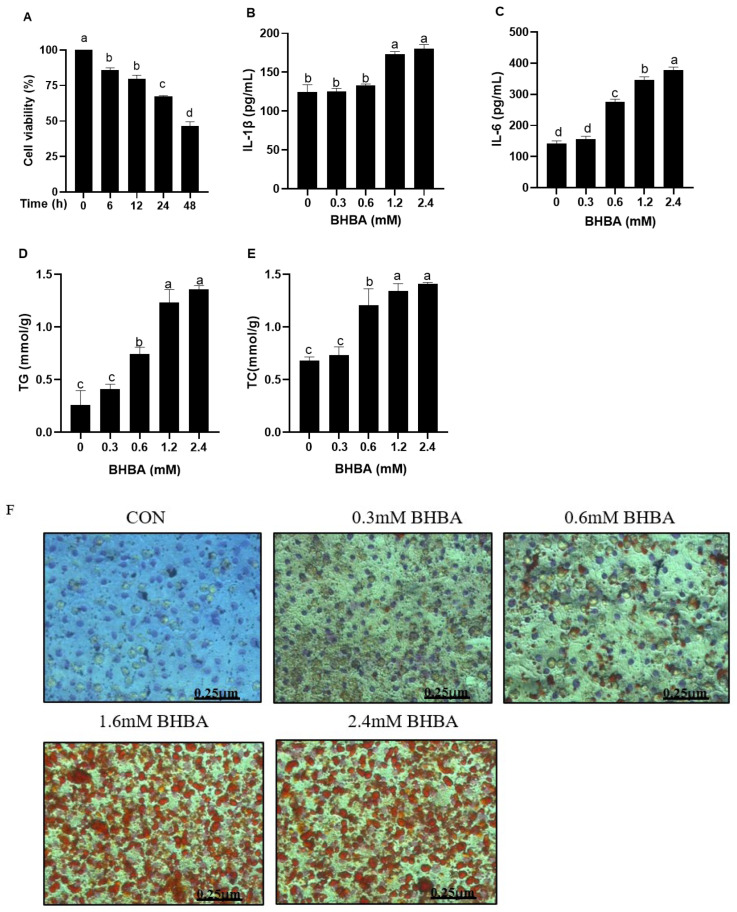
Effect of BHBA on hepatocyte viability, liver function, and lipid accumulation in calves. (**A**) The cell viability; cells were challenged with 1.2 mM BHBA for different times (0, 6, 12, 24, 48 h), respectively. Cell viability was measured using the CCK-8 assay. (**B**,**C**) Effect of BHBA on liver function indices. Contents of IL-1β and IL-6 in hepatocytes were measured using bovine ELISA assay kits. (**D**–**F**) Effect of BHBA on lipid accumulation. The contents of TG and TC were measured using biochemical kits. Accumulation of lipid droplets in hepatocytes observed by Oil Red O staining. Cells were treated with different concentrations of BHBA (0, 0.3, 0.6, 1.2, and 2.4 mM) for 24 h. Each experiment was repeated at least six times. The data are shown as the mean ± SEM. The values with different lowercase letters are significantly different (*p* < 0.05).

As shown in Figure 1A, BHBA reduced the relative viability of hepatocytes at different time points (*p* < 0.05), with the lowest viability observed at 48 h, significantly lower than at 6, 12, and 24 h (*p* < 0.05). At 24 h, the viability was significantly lower than at 6 and 12 h (*p* < 0.05), but significantly higher than at 48 h (*p* < 0.05). Based on these results, a 24 h exposure time was selected for the BHBA injury model. Compared with the blank control, both 0.3 and 0.6 mM BHBA had no significant effect on hepatocyte IL-1β content (Figure 1B, *p* > 0.05). In contrast, 1.2 and 2.4 mM BHBA significantly increased hepatocyte IL-1β and IL-6 contents (Figure 1B,C, *p* < 0.01).

### 3.2. Effect of BHBA on Lipid Accumulation in Calf Hepatocytes

Both 1.2 and 2.4 mM BHBA significantly increased TG and TC contents in hepatocytes compared with the blank control (Figure 1D,E, *p* < 0.01). However, there was no significant difference between the two groups (Figure 1D,E, *p* > 0.05). In Oil Red O staining (Figure 1F), the nuclei appeared blue, round, or oval, while lipid droplets surrounding the nuclei were stained orange or red. The red lipid droplets in the cytoplasm of hepatocytes treated with 1.2 and 2.4 mM BHBA were significantly darker and covered a larger area compared to those treated with 0, 0.3, or 0.6 mM BHBA. Based on these observations, 1.2 mM BHBA was selected as the optimal concentration for the injury model in this experiment.

### 3.3. Effect of BHBA on Inflammatory Factors in Hepatocytes and the Intervention of RT

**Figure 2:**
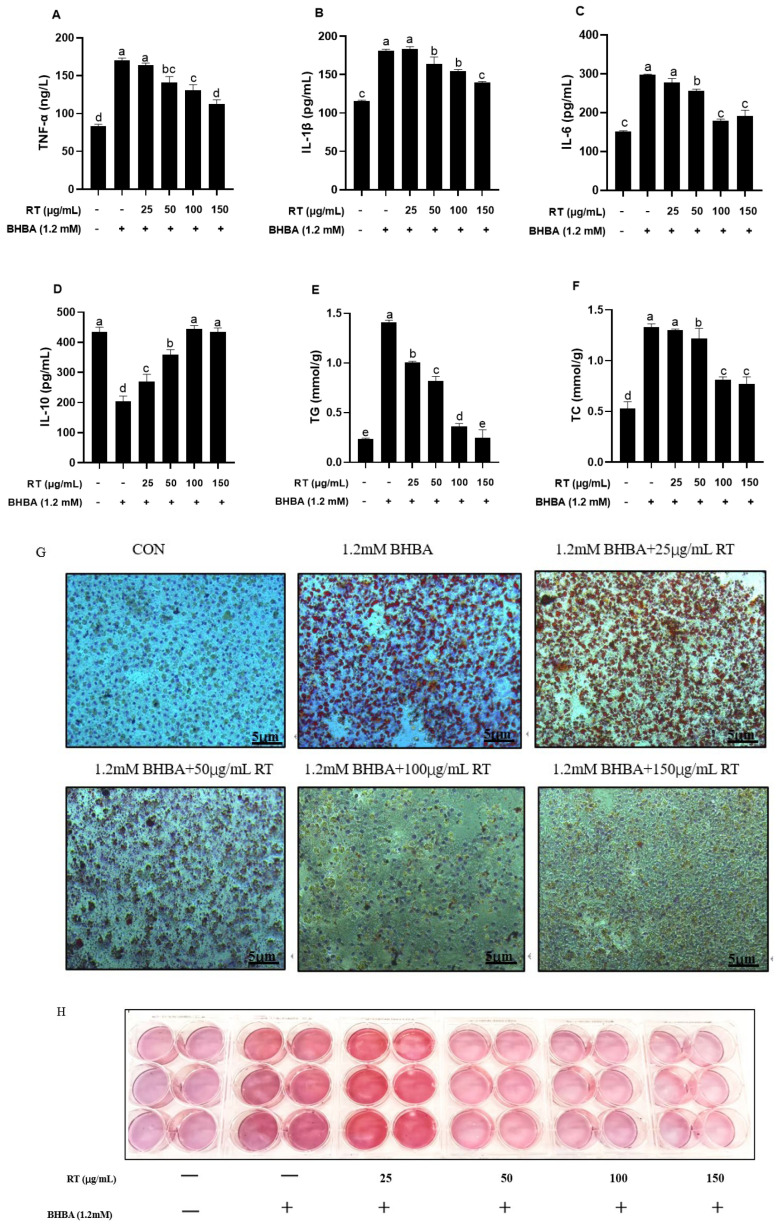
Effect of RT on BHBA-induced inflammation and lipid accumulation in calf hepatocytes. (**A**–**D**) The contents of TNF-a, IL-1β, IL-6, and IL-10 were measured using ELISA assay kits. (**E**–**H**) The contents of TG and TC were measured using biochemical kits. Accumulation of lipid droplets in hepatocytes observed by Oil Red O staining. Cells were pre-treated with different concentrations of RT (0, 25, 50, 100, 150 μg/mL) for 24 h and then challenged with 1.2 mM BHBA for 24 h. Each experiment was repeated at least six times. The data are shown as the mean ± SEM. The values with different lowercase letters are significantly different (*p* < 0.05).

As shown in Figure 2A–D, BHBA significantly increased TNF-α, IL-1β, and IL-6 contents, while it decreased the IL-10 content in hepatocytes (*p* < 0.01). These effects were reversed by pretreatment with different concentrations of RT, with 100 and 150 μg/mL demonstrating the most pronounced effect (*p* < 0.01).

### 3.4. Effect of RT on BHBA-Induced Lipid Accumulation in Hepatocytes

As shown in Figure 2E,F, compared with the BHBA treatment, groups pretreated with different concentrations of RT showed decreased TG and TC contents in hepatocytes. Hepatocytes pretreated with 100 and 150 μg/mL RT showed no significant difference in TC content between the two treatments. As shown in Figure 2G,H, Oil Red O staining revealed that the accumulation of red lipid droplets in hepatocytes was gradually reduced with increasing concentrations of rutin, with the most significant reduction observed at 100 and 150 μg/mL.

### 3.5. Effect of RT on the NF-κB Signaling Pathway

**Figure 3:**
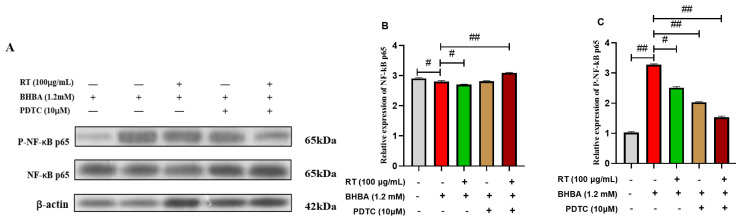

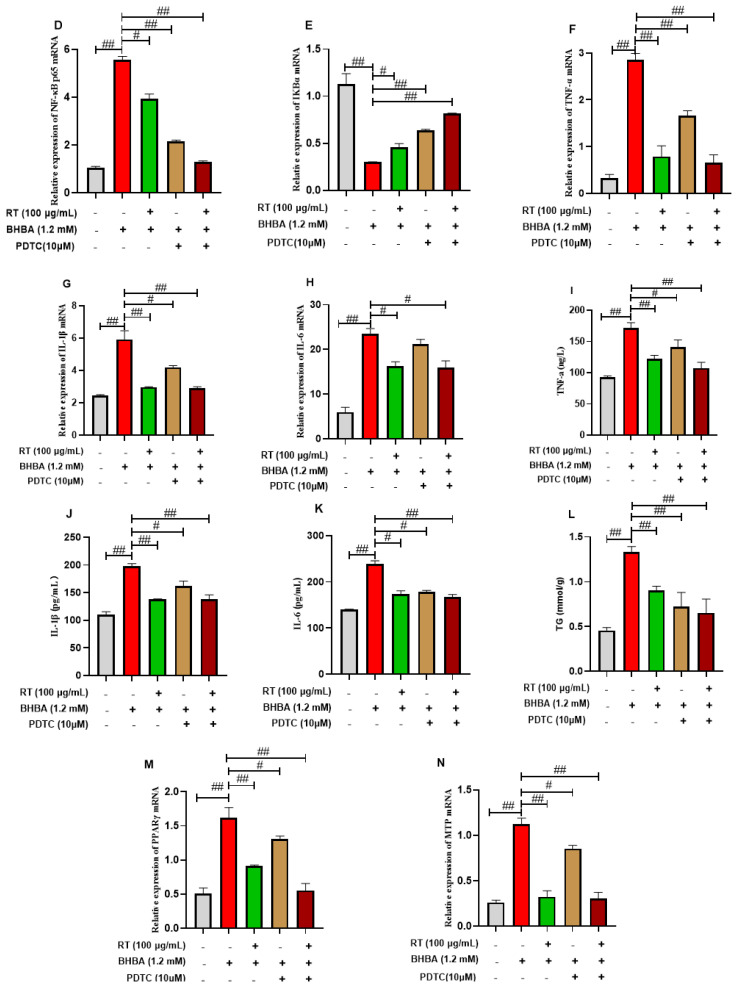
RT alleviated the inflammation and lipid accumulation induced by BHBA in calf hepatocytes by inhibiting NF-κB signaling pathway. (**A**–**C**) Expression levels of proteins NF-κB p65 and P- NF-κB p65 were detected using Western blotting, and β-actin was used as a control. (**D**–**H**) Relative mRNA expression levels of NF-κB p65, IKBα, TNF-α, IL-1β, and IL-6 were detected using RT–qPCR, with β-actin used as an endogenous control. (**I**–**K**) The contents of TNF-a, IL-1β, and IL-6 were measured using ELISA assay kits. (**L**) The content of TG was measured using biochemical kits. (**M**,**N**) Relative mRNA expression levels of PPARγ and MTP were detected using RT–qPCR, with β-actin used as an endogenous control. Hepatocytes were divided into five treatments: blank controls, BHBA treatment (cells were treated with 1.2 mM BHBA for 24 h), RT+BHBA treatment (cells were pre-treated with 150 μg/mL RT for 24 h and then challenged with 1.2 mM BHBA for 24 h), PDTC + BHBA treatment (hepatocytes were incubated with 10 μM NFκB inhibitor-PDTC for 2 h and then challenged with 1.2 mM BHBA for 24 h), or RT + PDTC + BHBA treatment (RT and PDTC were pretreated together and then treated with BHBA for 24 h). Each experiment was repeated at least six times. # means significant difference (*p* < 0.05); ## means highly significant difference (*p* < 0.01).

As shown in Figure 3A–E, compared with the blank control, BHBA highly significant downregulated the relative mRNA expression level of IκBα (*p* < 0.01). The protein expression level of P-NF-κB p65 and the relative mRNA expression of NF-κB p65 were highly significant upregulated (*p* < 0.01). However, RT + BHBA treatment significantly reversed these phenomena compared with the BHBA treatment (*p* < 0.05). Likewise, compared with the BHBA treatment, the PDTC + BHBA treatment significantly downregulated the relative protein expression of P-NF-κB p65 and the relative mRNA expression of NF-κB p65 (*p* < 0.05). In contrast, the relative mRNA expression of IκBα was significantly upregulated (*p* < 0.05). Additionally, this was more pronounced after RT and PDTC pretreatment.

### 3.6. The Expressions of Pro-Inflammatory Factors in Hepatocytes

To determine whether RT ameliorates BHBA-induced hepatocyte injury via the NF-κB signaling pathway, we further assessed inflammatory cytokine expression and contents following treatment with the NF-κB inhibitor PDTC. As shown in Figure 3F–H, in the qRT-PCR assay, compared with the BHBA treatment, the relative mRNA expression levels of TNF-α, IL-1β, and IL-6 in the RT + BHBA, PDTC + BHBA, and RT + PDTC + BHBA treatments were downregulated, especially in the RT + PDTC + BHBA treatment (*p* < 0.05). ELISA was performed to assess the contents of TNF-α, IL-1β, and IL-6 between treatments, and the results were consistent with those described above.

### 3.7. The Expressions of Lipid Metabolism Genes in Hepatocytes

To determine whether RT ameliorates BHBA-induced lipid metabolism injury in hepatocytes via the NF-κB signaling pathway, we further assessed lipid metabolism gene expression and contents following treatment with the NF-κB inhibitor PDTC. In the biochemical kits assay, the addition of PDTC inhibitor alone significantly reduced hepatocyte TG content compared with the BHBA treatment (Figure 3L, *p* < 0.05). This was more pronounced after pretreatment with RT combined with PDTC inhibitor. In the qRT-PCR assay, compared with BHBA treatment, hepatocytes pretreated with either RT or PDTC showed significantly downregulated relative mRNA expressions of PPARγ and MTP (Figure 3M,N, *p* < 0.05). This effect was more pronounced when RT and PDTC were combined.

## 4. Discussion

The prevalence of SCK in dairy cows is approximately 30%, and it is often undiagnosed, leading to significant economic losses for farms. During this period, cows experience NEB, resulting in disordered lipid metabolism and an excessive production of BHBA, which causes liver damage [5,17]. RT, a natural, environmentally friendly plant extract with stable biological activity, possesses various pharmacological effects [18]. However, research on the use of RT in ruminants is limited. This study aimed to investigate the effects of different BHBA concentrations on calf hepatocytes and to explore the mechanism by which RT alleviates BHBA-induced inflammation and lipid metabolism injury in hepatocytes.

This study found that after 48 h of BHBA exposure, calf hepatocyte viability was significantly reduced, negatively impacting cell growth and making further experiments unsuitable. Cell viability at 24 h was significantly lower than at 6 and 12 h but higher than at 48 h, establishing 24 h as the optimal time point for the BHBA-induced injury model. This is consistent with findings by Zhou [19]. IL-1β and IL-6 are typical pro-inflammatory factors, and when lipid damage occurs in hepatocytes, the NF-kB signaling pathway is activated, accompanied by elevated contents of pro-inflammatory factors [20]. Shi et al. [21] reported that 24 h exposure to high concentrations of BHBA activated the NF-κB signaling pathway in calf hepatocytes, significantly increasing pro-inflammatory cytokine levels. In this study, exposure to different BHBA concentrations led to elevated IL-1β and IL-6 contents in hepatocytes, with the most significant effects observed at 1.2 and 2.4 mM BHBA, indicating that high BHBA concentrations cause inflammatory damage to hepatocytes. TG and TC are key lipids that serve as important energy sources for the body [16], and with the liver being the primary site of their synthesis and catabolism, hepatocellular injury can disrupt their metabolism [22,23]. In this study, 24 h high-BHBA treatment caused a significant increase in TG and TC contents in calf hepatocytes. Oil Red O staining revealed significant lipid droplet accumulation in hepatocytes treated with high BHBA concentrations, further indicating that high BHBA levels disrupt lipid metabolism. Du et al. found that the expression levels of PPARγ and sterol regulatory element-binding protein 1c (SREBP1c) in the blood of SCK cows were significantly increased, while the expression levels of PPARα, carnitine palmitoyltransferase 1A (CPT1A), SREBP2, and 3-hydroxy-3-methylglutaryl coenzyme A reductase (HMGCR) were significantly decreased [15]. In vitro studies further confirmed that high concentrations of BHBA led to an increased expression of PPARγ and SREBP1c, while decreasing the expression of PPARα, CPT1A, SREBP2, and HMGCR in hepatocytes of dairy cows. This alteration in gene expression resulted in an increased triglyceride (TG) content in hepatocytes and a decrease in VLDL and LDL-C content [15]. Thus, BHBA primarily regulates these lipid metabolism-related genes, contributing to lipid accumulation and disorders in lipid metabolism in cow hepatocytes. Similarly, Zhou et al. showed that 4 mM BHBA treatment for 12 h significantly increased the TG content in calf hepatocytes [24]. Elshafey et al. found a positive correlation between TG and BHBA levels and the extent of hepatic lipid metabolism impairment in dairy cows with fatty liver [25]. In contrast, Deng et al. reported that while 0.6–2.4 mM BHBA induced lipid peroxidation in calf hepatocytes, it did not increase the TG content, which contradicts our findings [26]. This discrepancy may be due to the shorter exposure time in their study, which might not have been sufficient to induce TG accumulation.

BHBA has been reported to induce inflammation in the bovine mammary endothelial cells (BMECs) of dairy cows, leading to an increased expression of inflammatory markers, including TNF-α, IL-6, and IL-1β [27]. Kandemir et al. demonstrated that the oral administration of 50 or 100 mg/kg RT in mice reduced AST and AKP activities, as well as TNF-α and IL-6 levels in liver tissues, thereby alleviating liver injury and inflammation [28]. Similarly, Li et al. found that RT sodium supplementation in mice enhanced β-oxidation in adipose tissue, reduced lipid peroxidation in liver tissue, and significantly decreased TG content [29]. To determine whether RT could mitigate BHBA-induced inflammation and lipid accumulation in calf hepatocytes, we pretreated cells with different RT concentrations and measured inflammatory cytokine levels and lipid accumulation. Previous studies have demonstrated that RT at concentrations of 0, 25, 50, 100, and 150 μg/mL had no adverse effect on the viability of sheep rumen epithelial cells [30]. Luo et al. reported that RT within the same concentration range enhanced antioxidant capacity and inhibited apoptosis in bovine mammary epithelial cells to varying degrees [31]. Based on these findings, RT concentrations of 0, 25, 50, 100, and 150 μg/mL were initially selected in the present study to evaluate its pre-protective effects. Our results showed that 1.2 mM BHBA significantly decreased IL-10 contents while increasing TNF-α, IL-1β, IL-6, TC, and TG contents in hepatocytes. However, RT pretreatment alleviated BHBA-induced hepatic impairment and inflammation in a dose-dependent manner, with 100 or 150 μg/mL RT showing the most pronounced protective effects. Oil Red O staining further confirmed that BHBA caused significant lipid accumulation, while 100 or 150 μg/mL RT effectively mitigated this effect, demonstrating RT’s role in alleviating BHBA-induced lipid accumulation. However, a limitation of this study is that the potential effects of varying RT pre-treatment durations on hepatocyte injury were not explored.

The NF-κB signaling pathway is a major inflammatory pathway in the body [32,33]. Shi et al. demonstrated that high concentrations of BHBA significantly increased IKKβ activity and phosphorylated IκBα (p-IκBα) levels in calf hepatocytes, along with elevated p65 expression and transcriptional activity [21]. In addition, the expression of the NF-κB-regulated inflammatory cytokines TNF-α, IL-6, and IL-1β was significantly upregulated, a result confirmed by corresponding increases in their levels [21]. Zou et al. reported that 2.4 mM BHBA induced lipid hyperaccumulation and downregulated PPARα mRNA expression, a key gene in lipolytic metabolism [34]. Our study found that compared to the blank control, high BHBA concentrations significantly decreased IκBα mRNA expression, while significantly increasing the protein expression of P-NF-κB p65 and NF-κB p65 mRNA levels. Additionally, the mRNA expression of TNF-α, IL-1β, and IL-6 was upregulated. Moreover, BHBA treatment led to a significant increase in the mRNA expression of PPARγ and MTP. PPARγ, which has the opposite effect of PPARα, promotes lipid synthesis, as indicated by the upregulation of PPARγ mRNA expression [24]. To investigate whether RT alleviates BHBA-induced inflammatory injury and lipid accumulation in calf hepatocytes via the NF-κB signaling pathway, we pretreated cells with the NF-κB inhibitor PDTC. PDTC is a potent NF-κB inhibitor that suppresses IκB phosphorylation, thereby preventing the nuclear translocation of NF-κB and reducing the expression of downstream cytokines [35]. Numerous in vivo and in vitro studies have demonstrated that PDTC exerts potential therapeutic effects in various diseases by inhibiting inflammation, oxidative stress, and other related pathological processes [36,37]. The results show that compared to BHBA treatment alone, RT or PDTC pretreatment, either individually or in combination, regulated the expression of key NF-κB pathway genes and reversed the observed inflammatory response and lipid accumulation, with the combined RT and PDTC treatment yielding the most significant effect. These findings suggest that RT mitigates BHBA-induced hepatocellular damage by modulating the NF-κB signaling pathway. Similarly, Jiang et al. demonstrated that the flavonoid quercetin at 100 μg/mL could alleviate LPS-induced inflammatory damage in bovine mammary epithelial cells (BMECs), inhibiting the NF-κB signaling pathway [38]. Additionally, Guo et al. showed that natural flavonoid lignans could reverse the inflammatory response induced by *Staphylococcus aureus* in BMECs [39]. Liu et al. reported that RT attenuated LPS-induced inflammation in mouse muscle cells by blocking NF-κB activation [40]. Cao demonstrated that quercetin, the primary active component of RT, at 50 μg/mL, inhibited key NF-κB pathway gene expression, reduced pro-inflammatory factors such as TNF-α and IL-1β, and significantly alleviated inflammation in an in vitro mouse mammary epithelial cell model [41]. Jin et al. further showed that RT mitigated TBHP-induced inflammatory damage in HepG2 cells by downregulating NF-κB and NF-κB p65 protein expression [42]. These findings are consistent with the results of the present study. Furthermore, it was demonstrated Liu et al. reported that RT inhibited lipid anabolism-related genes, including fatty acid synthase (FASN), SREBP1, and stearoyl-CoA desaturase 1 (SCD1), as well as TNF-α and IL-6. In turn, this alleviated palmitic acid (PA)-induced lipid accumulation and inflammatory injury in HepG2 cells [43]. Prince et al. showed that RT reduced the very low-density lipoprotein (VLDL) content and 3-hydroxy-3-methylglutaryl coenzyme A (HMG-CoA) activity in rat liver tissues, thereby alleviating hepatic lipid accumulation [44]. The binding site of NF-κB has been identified in the promoter region of SREBP1 [45,46,47]. These findings suggest that the inflammatory signaling pathway may play a role in regulating lipid metabolism. In this study, RT effectively reversed the BHBA-induced changes in the relative mRNA expression of calf hepatocyte lipid metabolism genes, including PPARγ and MTP, as well as TG content. This effect was more pronounced when PDTC, an NF-κB inhibitor, was added. It is hypothesized that RT alleviates BHBA-induced lipid accumulation in hepatocytes by inhibiting the NF-κB signaling pathway. However, the exact mechanism through which RT regulates specific target genes within the NF-κB pathway remains to be further investigated.

## 5. Conclusions

In summary, the present study indicates that high concentrations of BHBA significantly increase inflammatory cytokine levels in calf hepatocytes, resulting in excessive lipid accumulation. RT alleviated BHBA-induced inflammation and lipid metabolism impairment by modulating the NF-κB signaling pathway. These findings provide a theoretical foundation for the potential application of RT in managing SCK in dairy cows. However, there are some limitations to this study, and in vivo experiments are needed in the future to further investigate the protective effects of RT on systemic damage in SCK cows.

## Figures and Tables

**Table 1:** Primer sequences (F = forward; R = reverse).

Gene	Accession No.	Primer Sequence (5′ to 3′)	Length (bp)
*ACTB*	NM_280979	F: GCAAATGCTTCTAGGCGGAC	203
		R: ATGCTCGATCCAACCGACTG	
*IKBα*	NM_348923	F: TGCAGGCCACCAACTACAAT	203
		R: GACATCAGCCCCACACTTCA	
*NF-κB p65*	NM_508233	F: CCAGACCAACAACAACCCCT	241
		R: CAGGAAGATCTCATCCCCGC	
*TNF-α*	NM_497021	F: TGCCTTGCTCAGATGTGTT	181
		R: GAGCGGAGGTTCAGTGATGT	
*IL-1β*	NM_370871	F: AAGCCACCCAGGGATCCTAT	199
		R: CCATCTCCCATGGAACCGAG	
*IL-6*	NM_378232	F: TGCAGGCCACCAACTACAAT	203
		R: GACATCAGCCCCACACTTCA	
*PPARγ*	NM_497014	F: GCCCCAGGTGGTGGTGGA	281
		R: GTAGGAAGTCTGCCGAGAGC	
*MTP*	NM_532992	F: GAGGGTGGATTTACGACCC	238
		R: GATGGCTGCAACCTGCTTTC	

## Data Availability

The data presented in this study are available within the article.
